# Comparison and Evaluation of Recombinant Antigens for ELISA-Based Diagnosis of *Schistosoma japonicum* Infection in Goats

**DOI:** 10.3390/microorganisms14102195

**Published:** 2026-10-01

**Authors:** Yilong Li, Mengge Xu, Kaijian Yuan, Ke Lu, Hao Li, Yan Zhou, Oyetunde T. Oyeyemi, Yang Hong, Xingang Yu, Zhiqiang Fu

**Affiliations:** 1 National Reference Laboratory for Animal Schistosomiasis, Key Laboratory of Animal Parasitology of Ministry of Agriculture and Rural Affairs, Shanghai Veterinary Research Institute, Chinese Academy of Agricultural Sciences, Shanghai 200241, China; 2 School of Animal Science and Technology, Foshan University, Foshan 528231, China; 3 National Key Laboratory of Intelligent Tracking and Forecasting for Infectious Diseases, National Institute of Parasitic Diseases, Chinese Center for Diseases Control and Prevention (Chinese Center for Tropical Diseases Research), Key Laboratory of Parasite and Vector Biology, National Health Commission of the People’s Republic of China (NHC), World Health Organization (WHO) Collaborating Center for Tropical Diseases, National Center for International Research on Tropical Diseases, Shanghai 200025, China; 4 Tropical Medicine & Diagnostics Development (TMDD) Group, Department of Biosciences and Biotechnology, University of Medical Sciences, Ondo City 351001, Nigeria

**Keywords:** *Schistosoma japonicum*, goat, ELISA, diagnosis, recombinant antigen

## Abstract

To address diagnostic challenges associated with caprine schistosomiasis under persistent transmission risks, this study evaluated the diagnostic performance of four recombinant antigens (rSjSAP4, rSj32, rSjCB1, and rSj23), with soluble egg antigen (SEA) serving as the reference comparator. An optimized indirect enzyme-linked immunosorbent assay (ELISA) was used to test serum samples from well-characterized goat panels, comprising 94 positive samples from goats experimentally infected with *Schistosoma japonicum* and 100 negative samples. Of the four recombinant antigens evaluated, rSjCB1 demonstrated the highest sensitivity (97.87%), whereas rSjSAP4 exhibited the highest specificity (98.00%). Receiver operating characteristic (ROC) curve analysis yielded Area Under the Curve (AUC) values of 0.8497, 0.9895, 0.8928, and 0.8050 for rSjSAP4, rSjCB1, rSj32, and rSj23, respectively. A mixture containing equal concentrations of rSjCB1 and rSjSAP4 achieved balanced performance (sensitivity: 93.62%, specificity: 92.00%; AUC = 0.9472) and excellent agreement with the reference standard (κ = 0.86). In conclusion, rSjCB1 appears to be the most promising single diagnostic antigen, and an equal-concentration mixture of rSjCB1 and rSjSAP4 represents a practical diagnostic alternative to SEA for the development of rapid and accurate immunoassays for caprine schistosomiasis.

## 1. Introduction

Schistosomiasis is a parasitic disease caused by blood flukes of the genus *Schistosoma* and is classified as a neglected tropical disease [1]. The elimination of schistosomiasis has become a major public health priority in many countries worldwide [2]. In China, the primary etiological agent of schistosomiasis is *Schistosoma japonicum* (*S. japonicum*), which is capable of infecting a wide range of definitive hosts, including humans, livestock such as goats and cattle, and wild animals [3,4,5]. Although schistosomiasis transmission in China has been effectively controlled, substantial challenges remain in some lake and marshland regions. These challenges include difficulties in managing reservoir hosts (such as wildlife) and controlling snail intermediate hosts, resulting in an ongoing risk of epidemic resurgence [1,6,7]. The broad mammalian host range of *S. japonicum* further complicates the epidemiology and control of schistosomiasis in China. Notably, goats, the populations of which have been expanding rapidly in recent years within endemic areas, represent an important domestic reservoir. Their typical grazing and rearing practices increase exposure to contaminated environments, thereby contributing to high infection rates [8,9]. In endemic areas, the co-grazing of goats and cattle facilitates mixed infections with zoonotic parasites such as *Echinococcus* spp., increasing the disease burden [10]. Therefore, highly sensitive and specific diagnostic methods are urgently needed to detect caprine schistosomiasis.

For the diagnosis of goat schistosomiasis, traditional methods such as fecal egg examination are labor-intensive and time-consuming, making them unsuitable for large-scale screening. In recent years, serological diagnostic techniques have attracted increasing attention due to their high sensitivity and practicality. ELISA and immunochromatographic tests (ICTs) are widely used for the diagnosis and epidemiological surveillance of schistosomiasis [1,11]. These serological approaches have also clarified the prevalence, spatial distribution, and genetic population structure of other parasitic zoonoses in goats, such as *Toxoplasma gondii* [12]. Currently, the serological diagnosis of caprine schistosomiasis in China predominantly relies on soluble egg antigen (SEA), which remains one of the most commonly used diagnostic antigens for schistosomiasis [3,13]. Xu et al. [13] developed a SEA-based colloidal gold immunochromatographic assay for detecting *S. japonicum* infection in livestock [13]. However, SEA preparation has several limitations, including laborious preparation, difficulty in quality control, and complex antigenic composition. In contrast, recombinant proteins offer several advantages, including simpler production, easier standardization, and well-defined components. Therefore, diagnostic methods based on recombinant proteins should be further developed and systematically evaluated.

Extensive immunological research on *S. japonicum* has led to the identification of a growing number of promising immunodiagnostic targets [14,15]. Given the variations in experimental conditions and interspecies differences between humans and livestock, a systematic comparative evaluation of these candidate targets is essential [14]. For instance, Sj23, a member of the tetraspanin family, is expressed in the tegument of *S. japonicum* with a molecular weight of approximately 23 kDa. It is recognized as an important candidate target for the development of both vaccines and diagnostic tools for schistosomiasis [15,16]. SjSAP4 has recently been identified as an important biomarker for immunodiagnosis, demonstrating excellent diagnostic performance for *S. japonicum* infection in the Philippines in human schistosomiasis [14,17,18]. Sj32 is a cysteine protease secreted by *S. japonicum*, playing a crucial role in host hemoglobin digestion and immune evasion. Previous studies have demonstrated its strong immunogenicity, eliciting robust immune responses in humans and other mammalian hosts [19,20]. SjCB1 is a schistosome cathepsin protease involved in nutrient acquisition, metabolism, and host invasion. It is expressed throughout all life stages of the parasite and is crucial for schistosome growth and development, and exhibits strong immunogenicity [21,22,23].

In this study, recombinant Sj23, SjSAP4, Sj32, and SjCB1 proteins were expressed using a prokaryotic expression system. The diagnostic performance of these four recombinant antigens was then evaluated for goat schistosomiasis by ELISA compared with SEA.

## 2. Materials and Methods

### 2.1. Parasites and Animals

*S. japonicum* cercariae were provided by the National Reference Laboratory for Animal Schistosomiasis, Shanghai Veterinary Research Institute, Chinese Academy of Agricultural Sciences. Six-week-old specific-pathogen-free (SPF) BALB/c mice were purchased from Shanghai Jiesijie Experimental Animal Co., Ltd (Shanghai, China). Each mouse was percutaneously infected via the abdominal skin with 40 cercariae for 20 min. At 42 days post-infection, mouse livers were collected for SEA preparation.

### 2.2. Sample Collection

A total of 94 serum samples were collected from goats experimentally infected with 300 *S. japonicum* cercariae. Infection was definitively confirmed by necropsy and worm perfusion, complemented by the fecal hatching test, and these samples were used as true positive specimens. One hundred (100) serum samples were collected from goats reared on a farm in a schistosomiasis non-endemic area in Weihai, Shandong Province, China. These samples were used as negative samples for *S. japonicum*. Twenty-four standard negative control serum samples were obtained from two-month-old healthy lambs at a farm in Nantong, Jiangsu Province, and they were used to set up the cutoff value. Standard positive and negative serum samples were prepared by pooling multiple strongly positive samples and multiple negative samples, respectively, from the laboratory stock. In addition, nine serum samples positive for *Haemonchus contortus* obtained from the Inner Mongolia Academy of Agricultural & Animal Husbandry Sciences were included. All serum samples were stored at −40 °C until use.

### 2.3. Preparation of Diagnostic Antigens

SEA of *S. japonicum* was prepared according to previously described methods [13,24]. Livers from mice infected for 6 weeks were homogenized in 1.2% NaCl solution. The homogenate was sequentially passed through stainless steel sieves of 40, 80, 120, 160, and 325 µm mesh size. The egg-containing eluate was passed through a 260 µm nylon mesh to collect the eggs, which were subsequently suspended in pre-chilled 1.2% NaCl solution. The suspension was aliquoted into 50 mL centrifuge tubes and centrifuged at 1575× *g* for 5 min. The supernatant was removed, and the pellet was resuspended in PBS containing 1 mg/mL collagenase type IV. Digestion was performed on a shaker at 37 °C for 1–2 h until the solution became clear with minimal sediment. After digestion, the mixture was centrifuged, and the supernatant was discarded. The resulting egg pellet was frozen at −80 °C, then ground under liquid nitrogen. The powder was resuspended in an appropriate volume of PBS and subjected to sonication at 30% power for 5 s with 55 s intervals, repeated 10 times. Finally, the suspension was centrifuged at 11,500× *g* for 20 min. The collected supernatant was used as SEA, which was aliquoted and stored at −80 °C.

All recombinant DE3 strains containing the plasmids rSj32-pET28a [20], rSjCB1-pcoldI [23], rGST-Sj23-pGEX [25], and rSjSAP4-pET21a were maintained at the National Reference Laboratory for Animal Schistosomiasis. The recombinant proteins rSjSAP4, rSj32, rSjCB1, and rSj23 exhibited molecular masses of approximately 22 kDa, 43.1 kDa, 32.3 kDa, and 31.7 kDa, respectively. The recombinant proteins were prepared using a similar protocol, as follows. Bacterial strains were revived from −80 °C storage, expanded in large-scale cultures, and induced with isopropyl β-D-1-thiogalactopyranoside (IPTG) to express recombinant proteins. The bacterial pellets were harvested by centrifugation, resuspended in 1× binding buffer, and lysed by repeated freeze–thaw cycles followed by sonication to produce recombinant protein-containing lysate. The recombinant protein rSj23 was purified under native conditions using GST•Bind Resin according to the manufacturer’s instructions (Merck, Darmstadt, Germany). In contrast, the recombinant proteins rSj32, rSjCB1, and rSjSAP4 were purified under denaturing conditions using Ni-NTA HIS•Bind Resin according to the manufacturer’s protocol (Merck, Germany). The purified recombinant proteins were subjected to 10% SDS-PAGE to confirm their expected molecular weights. All purified proteins required no refolding. Specifically, rSjSAP4, rSj32, and rSjCB1 were dialyzed against PBS containing 8 M urea, whereas rSj23 was dialyzed against PBS alone. Following concentration quantification using the bicinchoninic acid (BCA) assay, all protein aliquots were stored at −80 °C.

### 2.4. Antigenicity Analysis of Recombinant Proteins by Western Blotting

Purified recombinant protein samples, under denaturing or native conditions, were separated by 10% SDS-PAGE and transferred onto a 0.45 μm nitrocellulose (NC) membrane at 20 V for 15 min. The membrane was blocked with 5% (*w*/*v*) skimmed milk powder in Tris-buffered saline containing 0.05% Tween-20 (TBST) for 1 h and then washed three times with TBST. It was then incubated with goat anti-schistosome positive serum (diluted 1:100) at 4 °C overnight. After three washes with TBST, the membrane was incubated with horseradish peroxidase (HRP)-conjugated rabbit anti-goat IgG antibody (diluted 1:5000) at room temperature for 1 h. Following a final three washes with TBST, signals were detected using a chemiluminescence substrate according to the manufacturer’s instructions.

### 2.5. Diagnostic Performance Evaluation of Recombinant Proteins by ELISA

Purified recombinant proteins and SEA were quantified using a BCA Protein Assay Kit (Beyotime, Shanghai, China). Optimal ELISA conditions were determined by checkerboard titration.

Using an optimized ELISA protocol, we tested 20 positive, 20 negative, and 24 healthy lamb serum samples with either HRP-conjugated rabbit anti-goat IgG (Solarbio, Beijing, China) or HRP-conjugated Protein G (Sangon Biotech, Shanghai, China) as the secondary antibody. The results were compared to select the optimal secondary antibody for subsequent experiments.

Recombinant proteins and SEA were diluted to 1 μg/mL. Then, 100 μL of each diluted antigen was added per well of a 96-well plate, followed by overnight incubation at 4 °C. After washing, plates were blocked with 150 µL of PBST containing 1% gelatin for 1 h at 37 °C. Each incubation step was followed by three washes with 200 µL PBST for 5 min each. Color development was initiated by adding 100 µL/well of 3,3′,5,5′-tetramethylbenzidine (TMB) substrate. Following incubation at 37 °C for 15 min, the reaction was stopped with 150 µL/well of stop solution. The ELISA protocol for goat serum samples was similar. After checkerboard titration optimization, goat serum samples were used at a dilution of 1:200, and the selected secondary antibody was diluted according to its optimal dilution factor (100 µL/well). The Optical Density values at 450 nm (*OD*_450_) obtained from goat serum were used to calculate the sensitivity and specificity of each antigen. The two antigens exhibiting the highest sensitivity and specificity were then mixed in equal amounts and tested using the same goat serum panel to determine whether improved diagnostic performance could be achieved.

### 2.6. Data Analysis

All data were processed and analyzed using the spreadsheet software WPS Office (version 12.1.0.28022). Sensitivity and specificity were calculated as follows: Sensitivity = TP/(TP + FN), and specificity = TN/(FP + TN), where TP, FN, TN, and FP represent true positives, false negatives, true negatives, and false positives, respectively. Youden’s index = Sensitivity + Specificity − 1. Serum samples from healthy lambs were used as controls to calculate the mean (x) and standard deviation (SD) of *OD*_450_ values, and the cutoff value was defined as x + 3SD [26]. The kappa coefficient was calculated to assess the agreement between the ELISA results of serum samples and the corresponding true results [27].

All figures presented in the Results section, including scatter plots and ROC curves, were generated using GraphPad Prism (version 9.00).

## 3. Results

### 3.1. Expression and Purification of rSjSAP4, rSj32, rSjCB1 and rSj23

**Figure 1:**
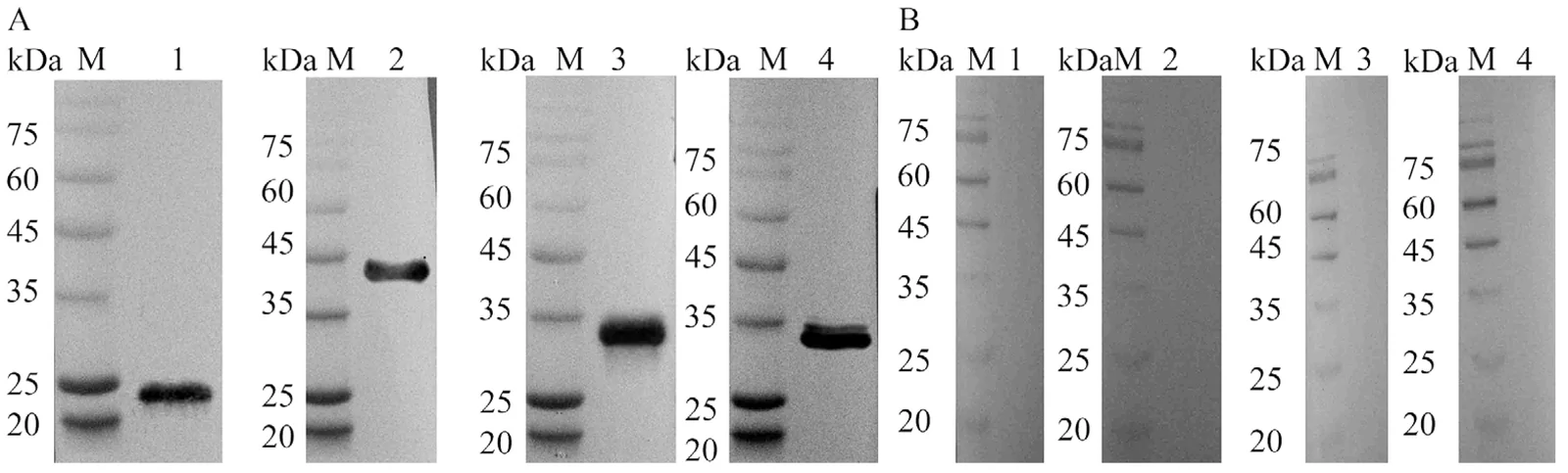
Western blotting analysis of the four recombinant proteins using goat sera. (**A**): positive serum; (**B**): negative serum; M, marker; lane 1: rSjSAP4; lane 2: rSj32; lane 3: rSjCB1; lane 4: rSj23.

The four recombinant proteins were expressed in *Escherichia coli*, with correct insert sequences verified by plasmid sequencing. Western blotting using standard goat sera (positive/negative) as primary antibodies revealed a single specific band for each recombinant protein (Figure 1).

### 3.2. Comparison of Secondary Antibodies for Detection

**Figure 2:**
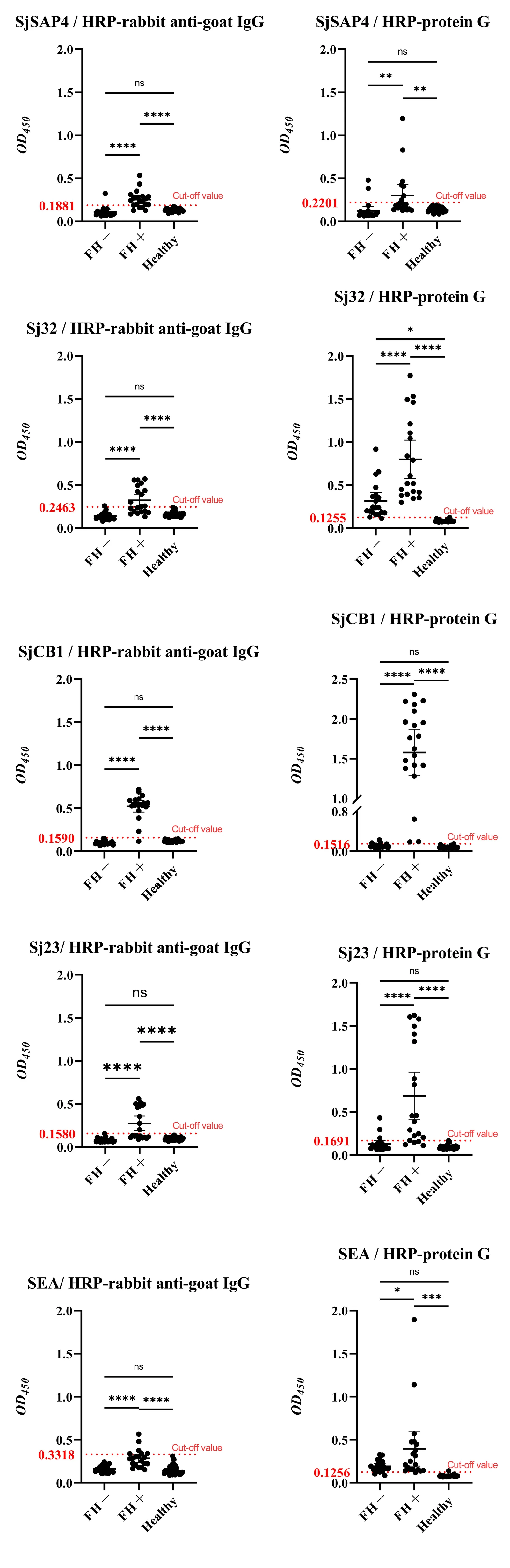
Comparison of detection performance between HRP-conjugated rabbit anti-goat IgG and HRP-conjugated Protein G using the four recombinant antigens and SEA (20 positive [FH+], 20 negative [FH−], and 24 healthy lamb sera). FH−, negative result in the fecal hatching test; FH+, positive result in the fecal hatching test. Each point in the figure represents a single data point. ns, not significant; * *p* < 0.05, ** *p* < 0.01, *** *p* < 0.001, **** *p* < 0.0001.

As shown in Figure 2, both secondary antibodies enabled a clear distinction in *OD*_450_ values between positive sera and negative goat sera or healthy lamb control sera. HRP-conjugated Protein G yielded higher OD values for positive sera, thereby reducing the likelihood of false-negative results. Although some false-positive reactions were observed with negative sera for certain antigens, particularly rSj32 and rSj23, HRP-conjugated Protein G was selected for subsequent ELISA experiments based on its overall detection performance.

### 3.3. Sensitivity and Specificity of ELISA for Goat Sera Based on the Four Recombinant Antigens and SEA

**Figure 3:**
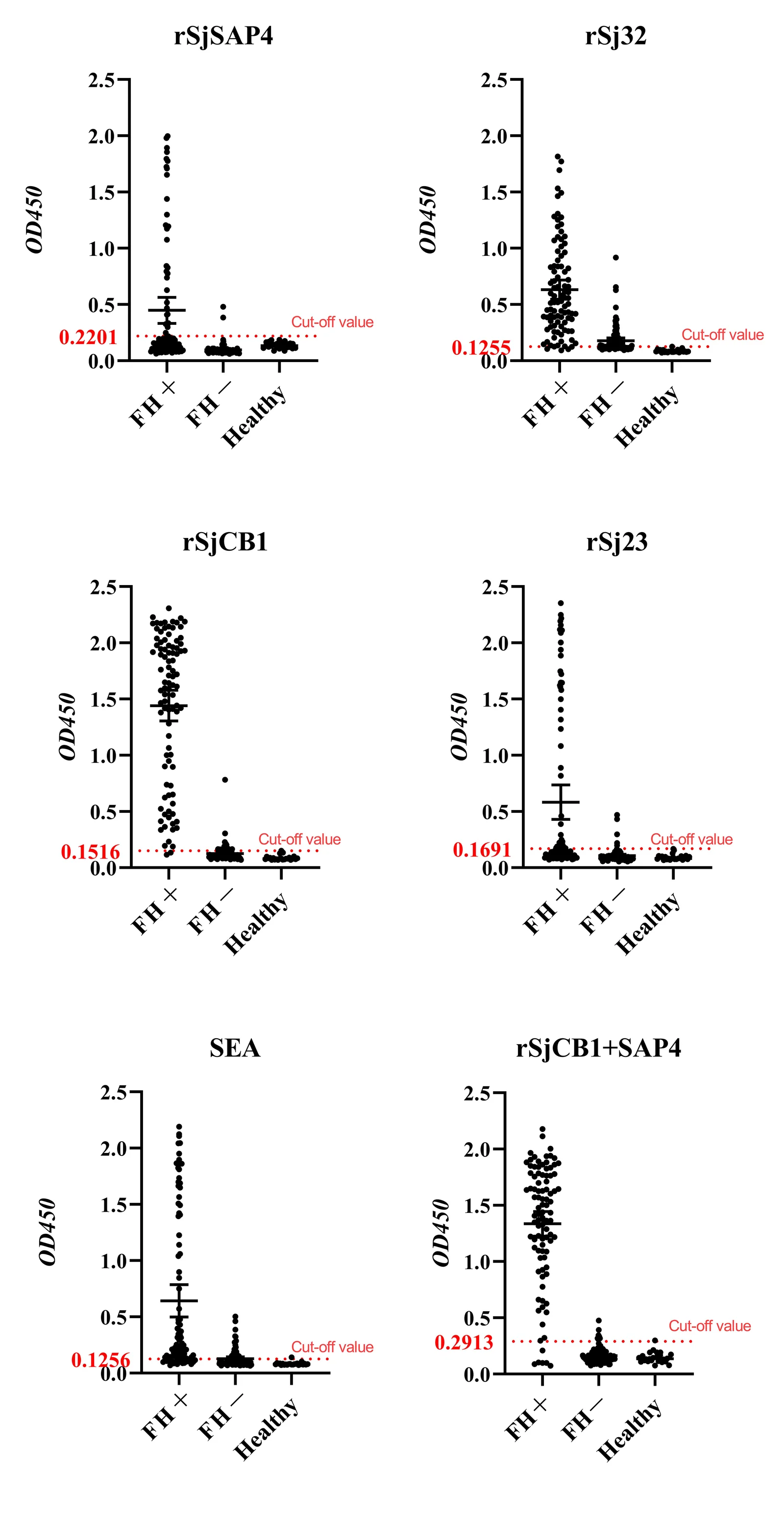
Distribution of *OD*_450_ values obtained by ELISA for 94 positive (FH+) and 100 negative (FH−) goat serum samples, as well as 24 healthy lamb sera, tested with 4 recombinant antigens and SEA. FH−: negative result in the fecal hatching test; FH+: positive result in the fecal hatching test. Each point in the figure represents a single data point.

Results of serum testing for anti-*Schistosoma japonicum* antibodies using recombinant proteins rSjSAP4, rSj32, rSjCB1, and rSj23, as well as SEA, as coating antigens are presented in Figure 3. The sensitivity and specificity of the four recombinant antigens and SEA, together with their 95% confidence intervals, were calculated and are presented in Table 1. Among the recombinant antigens, rSjCB1 showed the highest sensitivity at 97.87% (95% CI: 92.53–99.74%), while rSjSAP4 demonstrated the best specificity at 98.00% (95% CI: 92.96–99.76%). To achieve more balanced diagnostic performance, an equal mixture of rSjCB1 and rSjSAP4 was used to test the same panel of goat sera. The results, presented in Table 1, showed a sensitivity of 93.62% and a specificity of 92.00%. Compared with the individual recombinant antigens, the mixed-antigen approach provided a more balanced profile of sensitivity and specificity.

### 3.4. ROC Curve Analysis of ELISA Results Obtained Using Four Recombinant Antigens and SEA

**Figure 4:**
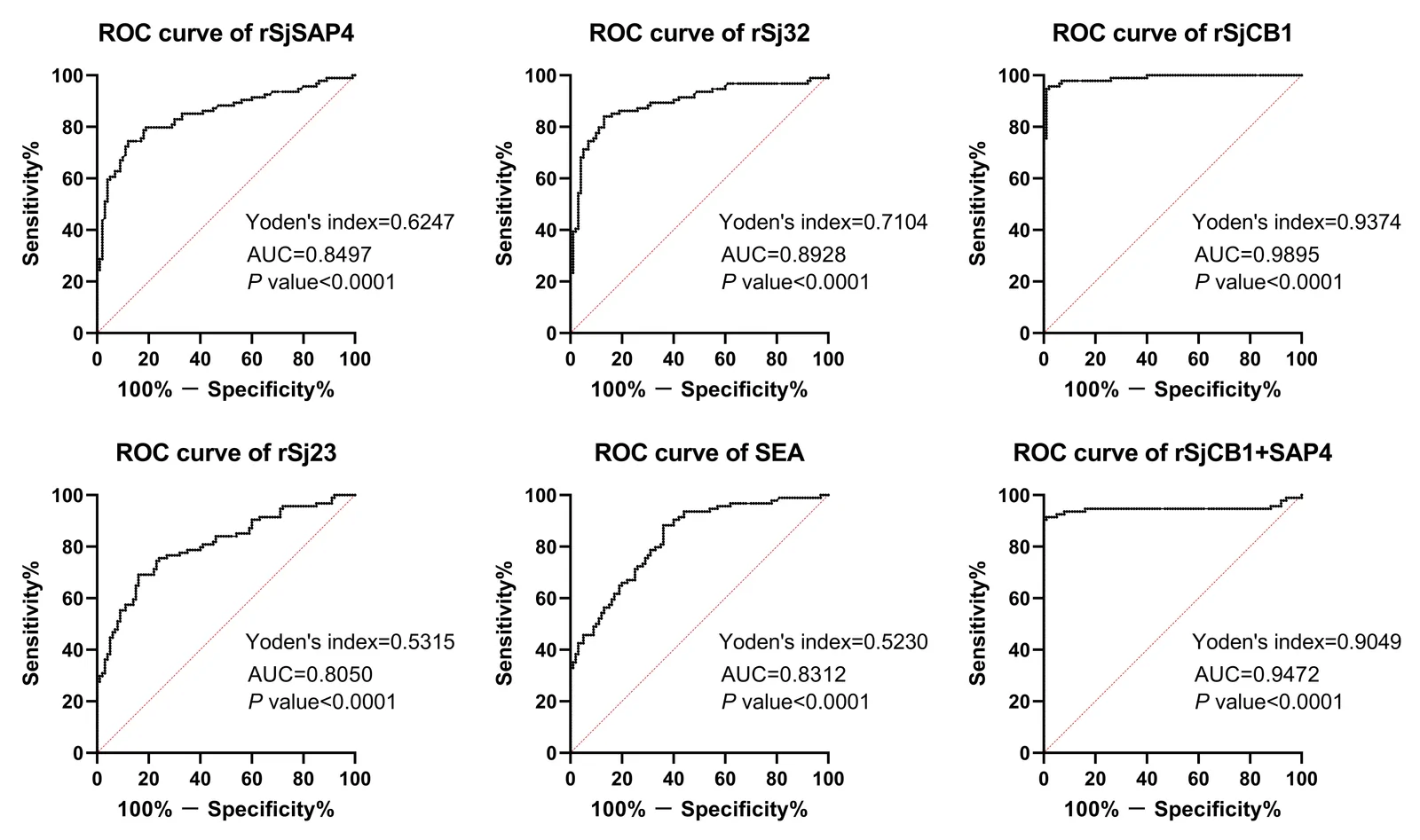
ROC curve analysis of the diagnostic performance of the four recombinant antigens and SEA using goat sera.

The results (Figure 4) showed that rSjCB1 had the largest area under the curve (AUC = 0.9895, 95% CI: 0.9781–1.000), demonstrating the highest diagnostic performance. This was followed by rSjSAP4, rSj32, with AUCs of 0.8497 (95% CI: 0.7934–0.9061) and 0.8928 (95% CI: 0.8445–0.9410), respectively, while rSj23 and SEA showed lower diagnostic potential, with AUCs of 0.8050 (95% CI: 0.7431–0.8669) and 0.8312 (95% CI: 0.7754–0.8870), respectively. The AUC obtained using an equal mixture of rSjCB1 and rSjSAP4 was 0.9472 (95% CI: 0.9048–0.9897), which was higher than that of SEA. Furthermore, Youden’s index was calculated for each antigen. rSjCB1 achieved the highest Youden’s index, at 0.9374 (0.8849–0.9794), followed by the equal mixture of rSjCB1 and rSjSAP4, with a value of 0.9049 (0.8411–0.9581) (Table 1).

### 3.5. Consistency Evaluation of the Four Recombinant Antigens and SEA for ELISA Test

The results (Table 2) showed that rSjCB1 had a kappa value of 0.78, indicating substantial agreement with the reference results. The kappa values for rSj23 and SEA were 0.42 and 0.48, respectively, reflecting moderate agreement. rSjSAP4 and rSj32 had kappa values of 0.38 and 0.37, respectively, indicating fair agreement. When rSjCB1 and rSjSAP4 were mixed in equal proportions, the kappa value increased to 0.86, demonstrating almost perfect agreement between the immunological and reference results.

### 3.6. Cross-Reactivity of Four Recombinant Antigens and SEA with Sera from Animals Infected with Other Helminths

To assess the cross-reactivity of the four recombinant antigens and SEA, ELISA was performed using nine serum samples positive for *H. contortus*. The results showed that all four recombinant antigens yielded weak positive reactions in 1–2 samples, whereas SEA showed no positive reactions (Table 3).

## 4. Discussion

Although substantial progress has been made in schistosomiasis control and prevention in China, the country is still in a critical stage in advancing the nationwide elimination of the disease [7,26]. Conventional parasitological methods, including fecal microscopy and the fecal miracidium hatching test, remain the commonly used reference approaches for diagnosing *S. japonicum* infection in animals. These conventional assays, however, exhibit markedly reduced sensitivity in low-intensity infections and prove impractical for large-scale field surveillance due to labor-intensive workflows, reliance on skilled technicians, and extended processing times [28,29]. Recombinant protein-based ELISA has proven to be a robust alternative: Wang et al. [30] developed a capsid protein-based ELISA for detection of PCV2 antibodies in swine serum, achieving 95.3% sensitivity and 93.9% specificity, further justifying the use of recombinant antigens in veterinary diagnostics. This applicability extends beyond viral pathogens, as Erguney and Aydın [31] successfully used a commercial ELISA to investigate *Neospora caninum* infection in cattle, demonstrating the platform’s utility for large-scale serological surveillance of livestock parasites.

Four candidate antigens (rSjSAP4, rSj32, rSjCB1, and rSj23) were selected for this study because they have been shown to induce strong humoral immune responses in mouse and bovine models of *S. japonicum* infection and may therefore serve as valuable diagnostic targets for schistosomiasis in goats [17,20,23,32]. Saposin-like proteins (SAP) are considered promising targets for schistosomiasis diagnosis [17], and their diagnostic application potential has also been confirmed in parallel comparative studies [14]. However, in the present study, SjSAP4 showed relatively low sensitivity but very high specificity when evaluated using the goat serum panel. Sj32 has also shown diagnostic potential for detecting *S. japonicum* infection in goat sera [20]. In this study, rSj32 produced high absorbance values and showed high sensitivity for the detection of positive goat sera. However, it also generated an unacceptably high false-positive rate among negative samples, resulting in markedly reduced specificity. This indicates that Sj32 may not be suitable for detecting anti-*S. japonicum* antibodies in goat serum. Cai et al. [33] used the recombinant Sj26GST-Sj32 fusion protein to detect IgG antibodies in sera from patients with chronic schistosomiasis japonica, reporting a sensitivity of 95.00% and a specificity of 97.67%. Fu et al. [20] previously used rSj32 for the serological diagnosis of *S. japonicum* infection in sheep and reported a sensitivity of 95.45% and a specificity of 85.71%. The observed differences in specificity may be attributable to differences in diagnostic criteria, experimental materials, sample size, and other factors. SjCB1 is a schistosome cathepsin B protease involved in nutrient acquisition, metabolism, and host invasion. It is expressed throughout all developmental stages of the parasite and plays a crucial role in its growth and survival, exhibiting favorable immunogenicity and antigenicity [21,22,23]. Since its identification by Bogitsh et al. [34] in 1983, numerous studies have focused on the functional characterization and expression patterns of SjCB. However, the diagnostic application of SjCB or SjCB1 has rarely been explored. In 2019, Macalanda et al. [35] evaluated SjCB1-based detection for human schistosomiasis japonica, reporting a sensitivity of 86.7% and a specificity of 96.7%. Given that SjCB1 is a key protein involved in host invasion and that SjCB1-based vaccines have demonstrated protective efficacy against *S. japonicum* infection [23], it is reasonable to hypothesize that SjCB1 possesses substantial diagnostic potential. The results of this study likewise indicate that SjCB1 has high diagnostic potential. Previous studies have shown that Sj23 antibodies perform well in the diagnosis of bovine schistosomiasis, with a sensitivity of 89.9% and a specificity of 91.2% [15,36]. Specifically, Lu et al. [25] used the rSj23 recombinant antigen for the serodiagnosis of *S. japonicum* infection in water buffalo and reported favorable diagnostic performance, while Li et al. [36] established an iELISA based on the recombinant LHD-Sj23 protein for detecting *S. japonicum* infection in cattle in China, demonstrating its utility in serological surveys. However, the recombinant antigen rSj23 showed low sensitivity (42.59%) and high specificity (95%) in ELISA-based detection of human schistosomiasis [14], a pattern consistent with the observations in this study. The diagnostic performance of the same antigen may differ among host species, which may be associated with interspecies differences in immune response characteristics, infection intensity, and other related factors. Collectively, these findings underscore the importance of evaluating recombinant antigens across different livestock host species, as antigen performance is host-dependent and cannot be reliably extrapolated from one species to another.

Notably, Youden’s index, AUC value, and kappa coefficient all indicated that SjCB1 had exceptionally high diagnostic potential for caprine schistosomiasis and might be superior to the traditional SEA. Macalanda et al. [35] applied this antigen in an ELISA for the diagnosis of human schistosomiasis and observed high sensitivity (86.7%) and specificity (96.7%), which is consistent with the results of the present study. Based on the comparison of the four recombinant antigens and SEA, the specificity of rSjCB1 required further optimization. Therefore, we focused on SjSAP4, which exhibited the highest specificity in this study, and has previously been reported to have diagnostic potential for detecting *S. japonicum* infection in the Philippines. Immunolocalization analysis revealed that SjSAP4 is predominantly expressed in the intestinal tract of adult worms [17], whereas SjCB1, a secreted cysteine protease associated with host tissue penetration, is highly abundant in schistosomal secretions [21,23]. Considering their distinct tissue distributions and complementary sensitivity/specificity profiles, we evaluated an equal mixture of rSjCB1 and rSjSAP4 on goat sera. While Youden’s index and AUC decreased marginally, the kappa coefficient rose to 0.86, indicating near-perfect agreement with true infection status. This synergy likely arises from the antigens’ divergent biological roles: SjCB1 (associated with early tissue invasion) and SjSAP4 (expressed in adult intestinal stages) capture distinct phases of antibody responses, while their diverse epitopes may help mitigate host-to-host immune variability. In this dual-antigen format, SjCB1 provides robust immunoreactivity, whereas SjSAP4 enhances specificity by curtailing cross-reactive false positives. Collectively, these complementary mechanisms render the rSjCB1+rSjSAP4 assay more balanced and reliable than rSjSAP4 alone; however, compared with rSjCB1 alone, each exhibits unique strengths across different evaluation frameworks (e.g., concordance vs. discriminative capacity). This multi-antigen strategy is consistent with previous reports. Bulashev et al. [37] demonstrated that combining multiple proteins improved diagnostic performance for bovine brucellosis compared with individual antigens. Similarly, Dang-Trinh et al. [38] showed that parallel evaluation of multiple antigen candidates enabled the selection of an optimized serological diagnostic strategy for schistosomiasis japonica. These findings collectively support the rationale that combining antigens with complementary biological roles and non-overlapping epitope profiles can achieve more balanced diagnostic performance than a single antigen alone, demonstrating certain advantages under different evaluation systems, yet its diagnostic potential relative to individual antigens remains to be further discussed. This principle of multi-antigen combination has gained traction beyond schistosomiasis: Bulashev et al. [39] demonstrated that an ELISA based on combined recombinant periplasmic proteins (BP26+SOD) achieved 91.6% sensitivity and 98.8% specificity for bovine brucellosis, outperforming individual antigens and supporting the broader applicability of antigen cocktail strategies in veterinary serodiagnosis.

Various criteria can be used to classify samples as positive or negative. When selecting the method for calculating the cut-off value, we referred to the approach used by Wu et al. [26], in which the cut-off was defined as the mean *OD*_450_ value plus three standard deviations based on healthy lamb sera tested with the four recombinant antigens and SEA. Tóth et al. [40] employed ROC analysis to evaluate test samples and determine the optimal diagnostic threshold from the ROC curve. This method may be more objective because it achieves the optimal balance between sensitivity and specificity for each antigen. Accordingly, we recalculated the detection results using the ROC method described by Tóth et al. [40]. We added the ROC-derived optimal cut-off values and the corresponding sensitivity/specificity values as Supplementary Table S1. Although this recalibration led to more balanced sensitivity and specificity for each antigen, the different criteria did not alter the overall results or conclusions of the present study. Furthermore, using standard negative sera to establish the cut-off value may align more closely with practical diagnostic applications. However, the selection of healthy lamb serum for cut-off determination is consistent with standard parasitological practices aimed at defining a conservative baseline for “naive” negativity. Lambs raised under controlled, parasite-free conditions represent a reliable source of unequivocally negative sera, minimizing the risk of including pre-exposed animals that might occur in field-collected adult goats. Regardless of the criterion adopted, rigorous validation of ELISA results remains paramount. Vujin et al. [41] similarly employed a virus neutralization test to confirm ELISA-positive SARS-CoV-2 antibodies in dogs, illustrating a two-tiered serological approach that strengthens the reliability of recombinant antigen-based ELISA.

Regarding cross-reactivity, the current study evaluated nine serum samples positive for *H. contortus* due to the high degree of homology between *H. contortus* and *S. japonicum*. We acknowledge that additional evaluation against other common goat parasites, including *Fasciola* spp., *Trichostrongylus* spp., *Moniezia* spp., and *Eimeria* spp., would provide a more comprehensive assessment of diagnostic specificity under field conditions. Future studies would benefit from expanding the cross-reactivity panel to include these parasites. This is particularly relevant, as recent epidemiological surveillance of beef and dairy cattle shows that co-infections with multiple zoonotic parasites are common in naturally grazed livestock [42]. Furthermore, the positive serum samples used in this study were derived from goats experimentally infected with a standardized dose of 300 cercariae. While this controlled design ensures a well-defined infection status, experimental infection with a uniform cercarial dose may result in relatively consistent antibody profiles. In contrast, natural infections in field settings may involve variable worm burdens, different parasite developmental stages, and potential co-infections, all of which could influence the diagnostic performance of the antigens evaluated.

Furthermore, it should be noted that these antibody-based serological methods are constrained by their exclusive reliance on experimentally infected goats, restricted cross-reactivity profiling, and a relatively modest validation cohort. Separately from these study-specific constraints, antibody-based serological methods, including the indirect ELISA used in this study, inherently cannot differentiate active infection from past exposure, as specific IgG antibodies against *S. japonicum* may persist in the host for months or longer, potentially leading to false positives in cured animals or low-endemic settings. Therefore, the antigens evaluated here—particularly rSjCB1 and the rSjCB1+rSjSAP4 combination—are recommended primarily for large-scale epidemiological screening and monitoring of elimination programs when used alongside parasitological methods, rather than for early acute diagnosis or individual treatment assessment. Despite these limitations, the high diagnostic performance demonstrated by these antigens at the serological level provides a promising foundation for future multi-marker diagnostic kits. To further improve the discrimination of active infection, subsequent studies could consider incorporating circulating antigen targets (e.g., CCA or CAA) or nucleic acid markers into the detection platform.

## 5. Conclusions

In this study, four recombinant antigens previously screened and validated in our laboratory were expressed and purified, and SEA was prepared to evaluate their diagnostic potential. Our findings indicate that rSjCB1 exhibits high diagnostic potential for detecting caprine schistosomiasis. Moreover, an equal mixture of SjCB1 and SjSAP4 also demonstrated substantial diagnostic value. Both SjCB1 alone and its combination with SjSAP4 showed high application potential for the detection and screening of *S. japonicum* infection in goat serum, but further field validation is warranted prior to widespread implementation.

## Figures and Tables

**Table 1:** Sensitivity and specificity of an ELISA incorporating the four recombinant antigens and SEA for the detection of antibodies in goat serum samples.

Antigen	Sensitivity (95% CI)	Specificity (95% CI)	Youden’s Index (95% CI)
rSjSAP4	39.36% (29.44–49.98%)	98.00% (92.96–99.76%)	0.6247 (0.5108–0.7279)
rSj32	95.75% (89.46–98.83%)	42.00% (32.20–52.29%)	0.7104 (0.6066–0.8043)
rSjCB1	97.87% (92.53–99.74%)	81.00% (71.93–88.16%)	0.9374 (0.8849–0.9794)
rSj23	48.94% (38.48–59.46%)	92.00% (84.84–96.48%)	0.5315 (0.4151–0.6472)
SEA	78.72% (69.07–86.49%)	69.00% (58.97–77.87%)	0.5230 (0.4098–0.6336)
rSjCB1 + rSAP4	93.62% (86.62–97.62%)	92.00% (84.84–96.48%)	0.9049 (0.8411–0.9581)

**Table 2:** Consistency evaluation of the four recombinant antigens or SEA immunological test and the reference results, interpreted by Cohen’s kappa coefficient (k: <0, Poor; 0–0.20, Slight; 0.21–0.60, Moderate; 0.61–0.80, Substantial; 0.81–1.00, Almost Perfect).

Results of Antigen ELISA Detection	Parasitological Positive	Parasitological Negative	Total	Kappa Coefficient
rSjSAP4 positive	37	2	39	
rSjSAP4 negative	57	98	155	
Total	94	100	194	0.38
rSj32 positive	90	58	148	
rSj32 negative	4	42	46	
Total	94	100	194	0.37
rSjCB1 positive	92	19	111	
rSjCB1 negative	2	81	83	
Total	94	100	194	0.78
rSj23 positive	46	8	54	
rSj23 negative	48	92	140	
Total	94	100	194	0.42
SEA positive	74	31	105	
SEA negative	20	69	89	
Total	94	100	194	0.48
rSjCB1 + rSjSAP4 positive	88	8	96	
rSjCB1 + rSjSAP4 negative	6	92	98	
Total	94	100	194	0.86

**Table 3:** Cross-reactivity of the Four Recombinant Antigens and SEA with Sera from Goats Infected with *Haemonchus contortus*.

Antigen	*Haemonchus contortus*		
	Total	Positive	Prevalence (%)
rSjSAP4	9	1	11.11
rSj32	9	1	11.11
rSjCB1	9	1	11.11
rSj23	9	2	22.22
SEA	9	0	0.0

## Data Availability

Raw data are available from the corresponding author upon reasonable request.

## Supplementary Materials

The following supporting information can be downloaded at: https://www.mdpi.com/article/10.3390/microorganisms14102195/s1, Table S1: Optimal cutoff values derived from ROC curve analysis.
