# Supplementary material for: Comparison and Evaluation of Recombinant Antigens for ELISA-Based Diagnosis of Schistosoma japonicum Infection in Goats
Source: Microorganisms. 2026 Oct 1;14(10):2195. doi: 10.3390/microorganisms14102195 (PMC13707446; doi:10.3390/microorganisms14102195)
Supplement: Supplementary file 1 [file microorganisms-14-02195-s001.zip › microorganisms-4459754-supplementary.pdf]

Table S1. The best theoretical data calculated based on the ROC curve

Antigen	Sensitivity (95% CI)	Specificity (95% CI)	Kappa value	Cut-off value
rSjSAP4	74.47%(95% CI 64.81-82.20)	88%(95% CI 80.19-93.00）	0.63	0.1112
rSj32	82.98%(95% CI 75.33-90.08)	87%(95% CI 79.02-92.24)	0.70	0.2489
rSjCB1	95.75%(95% CI 89.56-98.33)	98%(95% CI 93.00-99.64)	0.94	0.2310
rSj23	69.15%(95% CI 59.21-77.58)	84%(95% CI 75.58-89.90)	0.53	0.1198
SEA	88.30%(95% CI 80.25-93.34)	64%(95% CI 54.24-72.73)	0.52	0.1042
rSjCB1+rSjSAP4	91.49%(95% CI 84.10-95.62)	99%(95% CI 94.55-99.95)	0.91	0.4171
